# Autophagy in graves’ ophthalmopathy

**DOI:** 10.3389/fcell.2023.1158279

**Published:** 2023-04-14

**Authors:** Yu-Qing Chen, Lian-Di Gao, Yi-Lin Liu, Ya Shen, Jia-Le Diao, Wei-Hua Yang, Rui-Li Wei

**Affiliations:** ^1^ Department of Ophthalmology, Changzheng Hospital of Naval Medicine University, Shanghai, China; ^2^ Department of Nursing, Changzheng Hospital of Naval Medicine University, Shanghai, China; ^3^ Shenzhen Eye Hospital, Jinan University, Shenzhen, China

**Keywords:** graves’ ophthalmopathy, autophagy, inflammation, adipogenesis, glycosaminoglycan

## Abstract

Graves’ ophthalmopathy (GO) is an inflammatory autoimmune disease that affects the eyes. It can significantly alter the quality of life in patients because of its distinctive pathological appearance and the effect on vision. To date, the exact pathological mechanism of GO has not been explicitly discovered. However, several studies have associated autophagy with this disease. Autophagy is a catabolic process that helps maintain homeostasis in all organisms by protecting the cells and tissues from various endogenous and exogenous stress factors. Based on our results, patients affected with GO have comparatively elevated levels of autophagy, which critically affects the pathological mechanism of the GO. In this review, we have summarized the autophagy mechanism in the pathogenesis of GO.

## 1 Introduction

Graves’ ophthalmopathy (GO) is the most common extrathyroidal manifestation in Graves’ disease (GD) patients, and it is an autoimmune disease that causes inflammation and damages the extraocular muscles and orbital adipose tissues (Bahn, 2010). Exophthalmos, upper eyelid retraction, conjunctival edema, and periorbital fat are some of the typical symptoms of GO. Most severe cases also present with corneal ulcers, perforation, and compressive optic neuropathy (Bartalena et al., 2021). However, the overall pathological mechanism is very complicated and not yet clearly established. T cells, which are stimulated by antigens, migrate and multiply continuously during the initiation and progression of GO. This process produces effector T cells, such as CD4^+^ helper T cells, CD8^+^ T cells, and regulatory T cells (Treg cell) (Fang et al., 2018). Interleukin-2 (IL-2), interferon-γ (INF-γ), and tumor necrosis factor (TNF) are all secreted primarily by type-1 helper T (Th1) cells during the early stages of GO, thereby promoting the progression from acute to chronic inflammation. The production of IL-4, IL-10, and auto-antibodies are all stimulated by type-2 helper T (Th2) cells, which predominate in the later stages of GO (Aniszewski et al., 2000). Research on the cytokines in GO indicates over-expression of Th1-like cytokines, including IL-1β, TNF-α, INF-γ, and IL-6, which are macrophage-derived. (Hiromatsu et al., 2000; Kumar and Bahn, 2003). Past studies have associated the primary pathological mechanism of GO with inflammation (Jang et al., 2016), adipogenesis (Longo and Higgins, 2019; Schrijver et al., 2019; Ko et al., 2021), and glycosaminoglycan (GAG) accumulation (Yoon et al., 2020). It has been suggested orbital fibrocytes (OFs) are the primary effector cell in GO (Wang and Smith, 2014). OFs contribute to orbital inflammation by proliferating and differentiating into myofibroblasts and adipocytes, producing excessive adipogenic factors and GAGs, and engaging in active crosstalk with macrophages and monocytes through chemokines and cytokines (Lehmann et al., 2008; Kuriyan et al., 2013).

Very few studies have related it to autophagy, a catabolic pathway that balances the degradation and synthesis of intracellular substances in almost all organisms. Autophagy is also associated with growth, development, and homeostasis at both the cellular and organismal levels (Morgan-Bathke et al., 2013; Fernández-Albarral et al., 2021). Under physiological conditions, appropriate levels of autophagy can help maintain cellular and organismal homeostasis. However, abnormal conditions, such as the production and accumulation of large amounts of inflammatory cytokines and excessive oxidative stress can lead to either insufficient autophagy or aggressive autophagy, which, in turn, can contribute to the pathogenesis of several different diseases (Yu et al., 2018). Thus, autophagy is a double-edged sword, with both positive and negative effects on the body. Autophagy is essential for the degradation of dysfunctional organelles and substances. However, autophagy can trigger programmed cell death that is not apoptotic if activated in an unchecked fashion (Mizushima and Levine, 2020).

In this review, we have focused on the aberrant role of autophagy in the development of GO pathogenesis. Excessive orbital inflammatory mediator, adipogenesis, and hydrophilic GAG deposit (including hyaluronic acid; HA) have all been linked to the pathological process of GO, as demonstrated in previous studies (Zhang et al., 2009; Yoon et al., 2015; Bifulco and Ciaglia, 2016; Li et al., 2018; Guo Y. et al., 2020; Li et al., 2021).

## 2 Overview of autophagy

Macroautophagy, chaperone-mediated autophagy, and microautophagy are the three types of autophagy. Cargo delivery into the lysosome (main autophagic organelle) can be classified into several categories based on the approaches followed. Among them, macroautophagy is the most dominant autophagy-regulation mechanism responsible for both external and internal environmental and physiological stimuli (Yang et al., 2019). Various factors, such as hypoxia, nutritional deficiencies, or infections, can induce autophagy. Therefore, cells need to regulate the autophagy levels appropriately to maintain the tissue and intracellular homeostasis. Autophagy participates in several processes that are vital for cell survival, such as removing altered organelles, eliminating viruses and bacteria, and preventing the accumulation of abnormal proteins (Mizushima and Klionsky, 2007). Indeed, past studies have shown that deleting the regulators of autophagy causes a significant accumulation of damaged organelles and proteins and increases the level of reactive oxygen species (ROS), which damage the various cellular components (Hara et al., 2006; Komatsu et al., 2006). Moreover, autophagy can affect the immune response by stimulating cytosolic antigen presentations regulated by major histocompatibility complex class II (MHC II) and compromising T and B cell homeostasis (Harris et al., 2009). Lysosomes also directly engulf the cytoplasmic materials due to microautophagy (Wang L. et al., 2022), a chaperone-mediated mechanism characterized by chaperone assistance in moving proteins, DNA, RNA, and other substrates across the lysosomal membrane (Kaushik and Cuervo, 2018). In this review, we primarily focused on the process and characteristics of macroautophagy.

### 2.1 Regulation of autophagy: Signaling pathways

**FIGURE 1:**
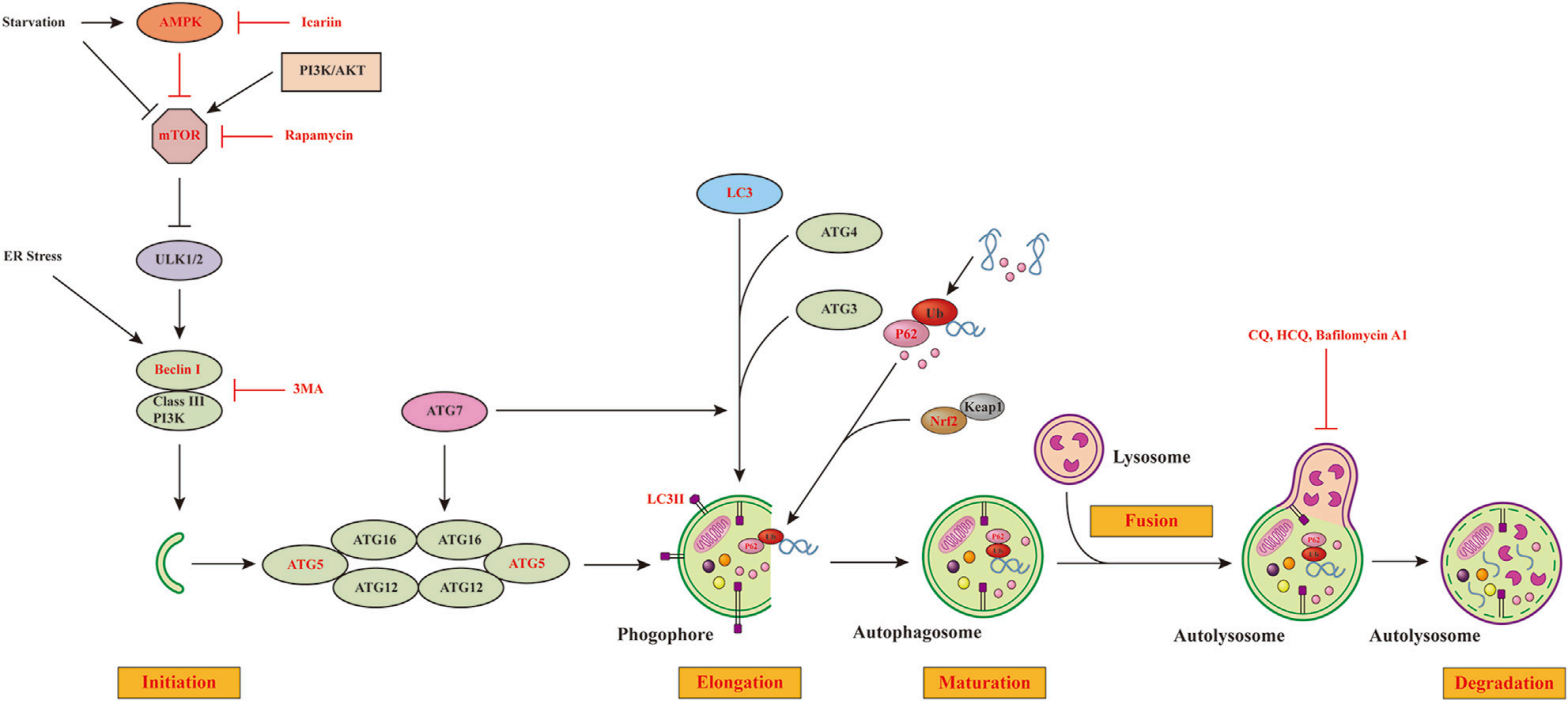
Major regulatory signaling pathways of autophagy. Autophagy is a process consisting of initiation, elongation, maturation and degradation steps. The PI3K-AKT pathway is the upstream activator of mTORC1, whereas AMPK initiates autophagy by inhibiting the mTORC1 activity or directly activating the ULK1/2 complex. mTORC1 activation inhibits autophagy by inhibiting the ULK1/2 complex, which is necessary for the induction of autophagy. When activating the ULK1/2 complex, it can recruit Beclin 1/III class PI3K complexes to the site of autophagosome formation. LC3-II exerts an essential role in the formation of autophagosome through binding to the autophagosomal membrane. P62 can serve as a connection between LC3 and ubiquitinated proteins. Autophagosome fuses with lysosome to form autolysosome. Eventually, autolysosome is degraded by lysosomal enzymes. We have highlighted in red the autophagic steps linked to inflammation, adipogenesis and HA accumulation in GO that have been discussed in this review. mTORC1, Rapamycin complex 1; ER, endoplasmic reticulum; rapamycin (mTOR) kinase; AMPK, AMP-dependent protein kinase; PI3K-AKT pathway, phosphoinositide 3-kinase (PI3K)–protein kinase B (AKT) pathway; ULK1/2 complex, UNC-51–like kinase1 or 2; LC3, light chain 3 protein; ATG, autophagy related genes; CQ, Chloroquine; HCQ, hydroxychloroquine.

Autophagy is a cellular process that involves various signaling pathways. Among these pathways, the mammalian target of rapamycin (mTOR), AMP-activated protein kinase (AMPK), phosphoinositide 3-kinase (PI3K)–protein kinase B (AKT), nuclear factor erythroid 2-related factor 2/Kelch-like ECH-associated protein 1 (NRF2/KEAP1), and endoplasmic reticulum (ER) stress pathways all play critical roles in coordinating autophagy (Figure 1). Autophagy can be activated by inhibiting mTOR signaling, whereas enhancing mTOR activity impairs it. In this regard, various growth factors and amino acids have been shown to activate the mTOR signaling pathway, while AMPK and p53 have been shown to inhibit it. Moreover, UNC-51–like kinase1 or 2(ULK1/2) phosphorylation is a downstream effector of activated mTOR that inhibits autophagy. AMPK activates autophagy by inhibiting mTORC1 signaling, as demonstrated by previous studies (Kim et al., 2011; Holczer et al., 2019; Li and Chen, 2019). When the cells are subjected to physicochemical irritations, NRF2/KEAP1 signaling is a crucial defense pathway against oxidative stress (Baird and Yamamoto, 2020). Some autophagy-deficient mice were found to have an abnormal accumulation of p62, which, in turn, led to an abnormal accumulation of NRF2 (Inami et al., 2011; Ichimura et al., 2013). Furthermore, p62 could inhibit KEAP1-mediated ubiquitination of NRF2 (Lau et al., 2010).

### 2.2 Pathological implications of autophagy

Autophagy is a fundamental process, and, like any other, it needs to be monitored to ensure a healthy equilibrium. Devastating pathologies in the body are triggered by disruptions in the autophagic pathways. Neurodegenerative disorders such as Alzheimer’s disease (Zhang et al., 2021), Parkinson’s disease (Lizama and Chu, 2021), and Huntington’s disease (Croce and Yamamoto, 2019), can all be triggered by impaired autophagy. As an essential housekeeping mechanism for maintaining energy homeostasis and cellular metabolisms, dysfunctional autophagy has been demonstrated to play the key role in the pathogenesis of cardiovascular diseases, including heart failure, atherosclerosis, cardiomyopathies, and ischemia-reperfusion injury (Wu et al., 2021). In several types of cancers, autophagy is crucial for cellular survival. It inhibits apoptosis, which helps induce tumor progression (Degenhardt et al., 2006; Cocco et al., 2020). Moreover, the homeostatic operation in the lung tissues requires functional autophagic reactions to maintain and ensure functional gas exchange. Dysfunctional autophagy has been linked to chronic obstructive pulmonary diseases (Yoshida et al., 2019) and pulmonary fibrosis (Zhao et al., 2020). In addition, aberrant autophagy has been linked to several eye diseases, such as cataracts (Zhou et al., 2016), glaucoma (Porter et al., 2013), age-related macular degeneration (ARMD) (Wang et al., 2009), diabetic retinopathy (DR) (Yao et al., 2014), and GO (Yoon et al., 2015). The interpretation of the underlying mechanisms linked with autophagy in ocular tissues and cells is of utmost significance and a crucial target in the potential therapeutic strategy (Maiuri et al., 2007).

## 3 The autophagy in GO

The CD34^+^ CD40^+^ orbital fibroblasts (OFs) initiate the pathogenesis of GO by activating helper T cells to recognize thyrotropin receptor (TSHR) peptides. Using the thyrotropin receptor antibody (TRAb), it forms a ligand for TSHR. This autoimmune reaction then stimulates the secretion of pro-inflammatory cytokines and increases GAG accumulation and adipogenesis in the periorbital tissues (Bahn, 2015). OFs can also express insulin-like growth factor I receptor (IGF-IR), with which TSHR can form physical and functional complexes. They can function synergistically to promote inflammation and activation of TSHR signaling and elevate the accumulation of HA. The activity of IGF-IR is an essential component in mediating the downstream signaling of TSHR. The inhibition of IGF-IR activity can reduce signaling initiated by either of the receptors**.** The induction of specific gene expression in fibroblasts and OFs by TSH can be attenuated with specific monoclonal antibodies to inhibit the activity of IGF-IR (Smith and Janssen, 2019). In recent years, an anti-IGF-IR antibody, teprotumumab, has been demonstrated to be efficient in alleviating several manifestations of TAO (Smith et al., 2017). Furthermore, the effects of the IGF-I/IGF-IR pathway on host immunity, tissue remodeling, and inflammatory regulation suggests its possible involvement in autoimmune diseases in addition to TAO. This finding has stimulated research into potential crossover components of the IGF-I pathway and other autoimmune disorders (Suzuki et al., 2015; Tsushima et al., 2017).

The pathogenesis of GO has been linked to inflammation, adipogenesis, and GAG accumulation, according to a few past studies (Jang et al., 2018). However, some other studies suggest that autophagy is only linked to GO. Notably, autophagy at an appropriate level can withstand a wide range of stresses, both endogenous and exogenous. For instance, the body can defend itself from infection, aging, hypoxia, and low energy by increasing the autophagy levels. However, the condition worsens and causes damage due to the extremely high level of autophagy (Jiang et al., 2019).

**FIGURE 2:**
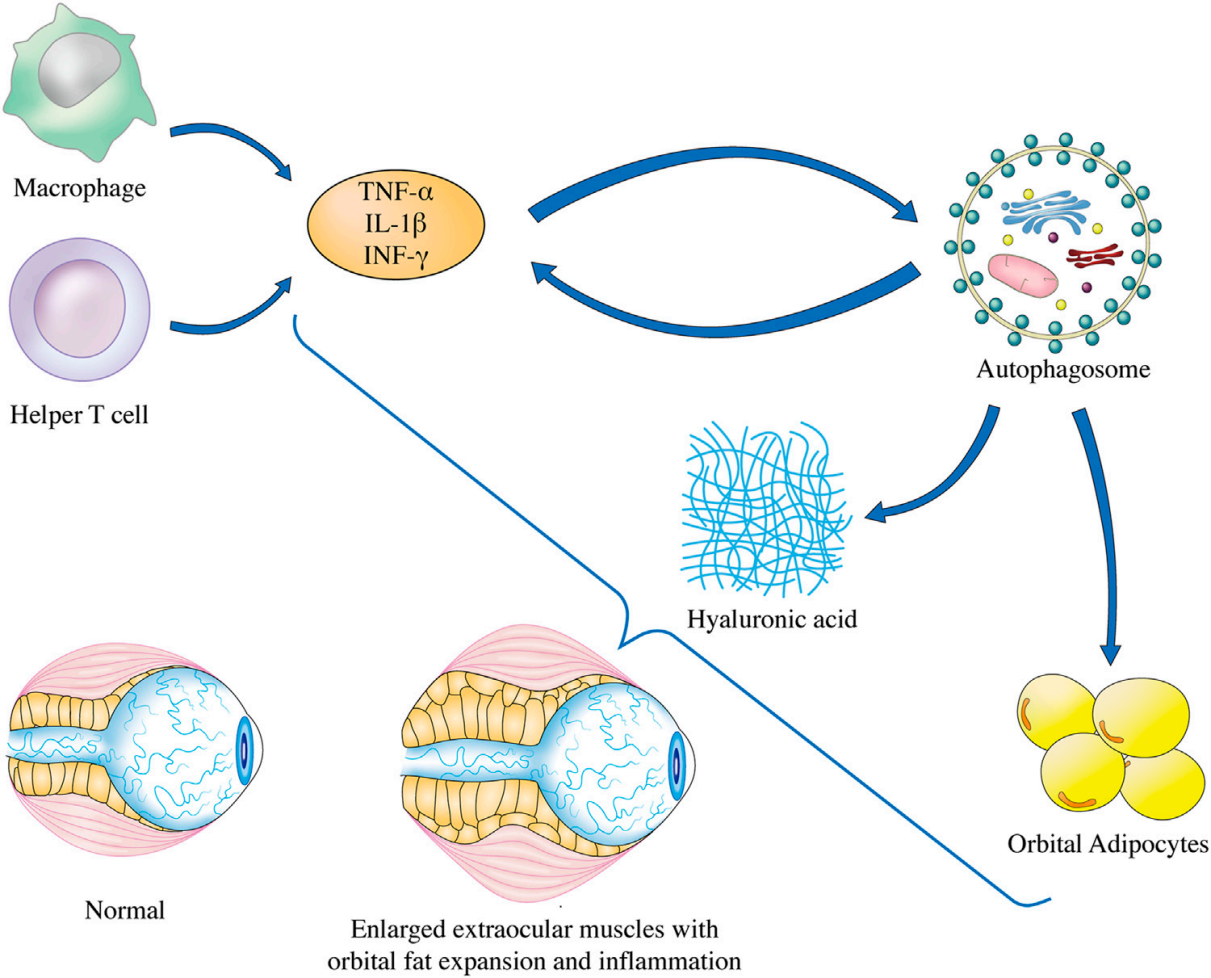
Autophagy regulation in GO pathogenesis. The underlying mechanisms of GO has been linked to inflammation, adipogenesis, and GAG accumulation in OFs, and finally lead to enlarged extraocular muscles with orbital fat expansion. Elevated secretion of inflammatory factors such as TNF-α, IL-1β, and INF-γ is associated with the induction of autophagy in OFs of GO. Progression of OFs adipogenesis can be mitigated by inhibition of autophagy. Furthermore, lysosomal inhibitors can reduce GAG accumulation through blocking the autophagic flux of OFs in GO. TNF-α, tumor necrosis factor-alpha; IL-1β, interleukin-1 beta; INF-γ, interferon-gamma.

It has been shown that OF, an effector cell, is indispensable in the pathogenesis of GO, and that it can participate actively in the remodeling of orbital tissues (Meyer zu Hörste et al., 2011). Orbital tissue modeling results from chronic fibrosis in the dormant stage of GO. At this stage, HA deposition and adipogenesis are possible. Thus, to slow the development of GO, inflammation, adipose tissue formation, and HA deposition must all be reduced, thereby making autophagy inhibition an essential therapeutic strategy (Potgieser et al., 2015). Inflammatory activity and oxidative stress can increase autophagy, which may ensure the survival of effector cells and contribute to GO pathogenesis. They can be triggered into an autoimmune pathological process by the primary pathogenic factors, as depicted in Figure 2.

### 3.1 Autophagy on inflammation in GO

OFs from GO patients can secrete inflammatory factors. There is a marked increase in the secretion level of these factors in GO patients when compared to those without GO. The inflammatory response of OFs may be exacerbated, and Th17 cells may be mobilized to further exacerbate GO as a result of their interactions with OFs (Fang et al., 2018). Thus, inflammatory processes are critically important in the pathogenesis of GO. Autophagy and inflammation have been linked by numerous researchers. The early stage of GO is marked by the interactions of infiltrating T cells with OFs, which causes increased cytokines and T cell activating factors (Lehmann et al., 2008). Suppressing orbital inflammation is a critical treatment in GO. Autophagy plays an important regulatory role through crosstalk with immune and inflammatory pathways, and aberrant autophagy underlies the pathogenesis of several inflammatory disorders (Ban and Tomer, 2003). Autophagy is essential for maintaining cellular homeostasis. The eye is an immunologically privileged organ (Niederkorn, 2006), and autophagy has been proved to be critical for the maintenance of eye immune privileges. Deletion of ATG5 in macrophages can cause uveitis, which, like GO, is also an autoimmune ocular disease. Inhibiting autophagy in macrophage can activate inflammasome-mediated IL-1β secretions in uveitis, and inhibitions of Caspase1 and Caspase4 completely reverse the disease phenotype (Santeford et al., 2016). Jaggi et al. (Jaggi et al., 2022) proved that blocking autophagy in M1 macrophage enhanced herpes simplex virus 1 replications in the eye, suggesting that modulating autophagy within macrophage may serve as a therapeutic pathway for ocular infections and inflammations. Macrophages are important in the intrinsic immunity of the eyes. Furthermore, macrophages are also indispensable in the pathogenesis of GO, producing a large number of pro-inflammatory factors, including IL-1β, TNF-α, and INF-γ(Kumar and Bahn, 2003). Interestingly, a study on the susceptibility of ATG5 variants to GD showed that the variant rs6937876 is located in ATG5 region and is closely related to susceptibility to GO (Wang W. et al., 2022). Li et al. (Li et al., 2018) demonstrated that IL-1β increased the mRNA levels of the inflammatory cytokines IL-6, IL-8, TNF-α, and MCP-1 in cultured OFs. The IL-1β-induced inflammation was concomitant with elevated autophagic activity, as manifested by elevated expressions of autophagy-associated proteins Beclin-1 and ATG-5 and conversions of LC3-I to LC3-II. Pre-treatment with the autophagic inhibitors 3-MA and bafilomycin A1, or silencing of the Beclin-1 and ATG-5, prevented IL-1β-induced inflammation in OFs, whereas pre-treatment with the autophagy activator rapamycin showed the opposite effect. These data suggested that autophagy was involved in GO and lead to orbital inflammation. In another study, Li et al. (Li et al., 2021) examined the effect of IL-13-induced autophagy on inflammation, ROS production and fibrosis, OFs derived from GO patients were treated with or without IL-13 and with or without the autophagy inhibitors 3-MA. The result showed that IL-13 treatment significantly upregulated TNF-α, IL-1β, and IL-6, but these effects could be partly reverted by 3-MA, suggesting that inflammation was correlated with the induction of autophagy in OFs of GO. Neferine is an alkaloid extracted from *Nelumbo nucifera*. It suppresses autophagy-mediated inflammation in OFs of GO, which may be moderated by the upregulation of Nrf2. It also elevates the LC3-II/LC3-I levels and reduces the p62 levels in OFs. The anti-inflammatory effect of neferine is correlated with the enhanced expression of Nrf2. Therefore, the proper control of autophagy has been associated with the attenuation of the orbital inflammation and alleviation in the progression of the GO, which makes this mechanism one of the potent therapeutic processes (Li et al., 2021). Moreover, it was found that neferine suppress autophagy through activating Nrf2 and PI3K/Akt/mTOR pathway in muscle cells (Baskaran et al., 2016). More evidence and mechanistic studies on the relationships between autophagy and inflammatory response in GO are still needed.

### 3.2 Autophagy on adipogenesis in GO

The level of proptosis in GO patients is largely determined by adipogenesis, which is an essential factor in its pathological process, as revealed by various studies. Nishida et al. (Nishida et al., 2002) found that the volume of the orbital tissue in GO patients is markedly larger than that in the non-GO ones. This study revealed a significant increase in the adipose tissues than that of the extraocular muscles. It has been hypothesized that adipose tissue’s cytokine-secreting capabilities contribute to the pathogenesis of GO (Ehrhart-Bornstein et al., 2003; Mimura et al., 2003; Park et al., 2020). Thus, it is evident that adipogenesis and OFs are critical for GO pathogenesis. It has been hypothesized that autoimmune activity leads to OF dysfunction (Bahn, 2003; Prabhakar et al., 2003) and that a subgroup of OFs can differentiate into mature adipocytes (Sorisky et al., 1996). GO patients have orbital muscle enlargement and fat expansion due to the overexpression of TSHR in mature adipocytes. Important factors in adipogenesis include growth arrest and the induction of transcriptional regulators; peroxisome proliferator-activated receptors *γ* (PPAR-γ) are also some of the pivotal factors (Rosen and MacDougald, 2006). Studies have demonstrated that autophagy is necessary for adipogenesis and can be inhibited by deleting the ATG7 gene to exert an anti-obesity action (Zhang et al., 2009). It has been observed that orbital adipogenesis could be activated by PPAR-γ agonists, which is accompanied by the TSHR upregulation in preadipocytes *in vitro* (Smith et al., 2002). In this regard, Yoon et al. (Yoon et al., 2015) observed that the autophagy levels were elevated in GO patients compared to that in the non-GO counterparts, indicating the involvement of autophagy in the pathogenesis of GO. This study demonstrated that inflammatory factors could induce autophagy. Moreover, it was shown that autophagosomes accumulated with lipid droplets in the GO tissues, thereby linking it with its pathogenesis. Furthermore, the treatment of Bafilomycin A1 and the knockdown of ATG5 expression via shRNA resulted in the inhibition of adipogenesis. The treatment of statins in such patients alleviated the progression of orbital fibroblast differentiation and adipogenesis by balancing the apoptosis and autophagy processes (Bifulco and Ciaglia, 2016). According to Li et al., icariin can inhibit the differentiation of preadipocytes into mature adipocytes by restoring the increases in LC3-II/LC3-I ratio; this effect is mediated by the inhibition of the AMPK/mTOR pathway (Li et al., 2017). Similarly, neferine was shown to inhibit autophagy-induced adipogenesis in OFs of GO, along with an upregulation of Nrf2 (Li et al., 2021). A previous study demonstrated that p62 inhibits adipocyte differentiation at early stages by blocking the basal ERK activity (Rodriguez et al., 2006).

### 3.3 Autophagy on GAG accumulation in GO

GAG accumulation is a pivotal process among all factors that lead to GO proptosis (Łacheta et al., 2019), and HA is the primary component of GAG (Guo J. Y. et al., 2020). The extraocular muscles of GO patients tend to swell (Zhang and Zhu, 2022) and pathological examination revealed that the swollen muscles are intricately subdivided by multiple amorphous particles primarily composed of GAG and collagen fibers (Smith et al., 1989). Additionally, due to its inherent hydrophilicity, the orbital fat and connective tissue can absorb water and result in edema. Chloroquine (CQ) and its derivative hydroxychloroquine (HCQ) are lysosomal inhibitors that prevent the degradation of autophagic substrates by blocking lysosomal acidification (Amaravadi et al., 2007). Both CQ and HCQ were found to decrease HA production by impairing the autophagic flux of GO-OF with or without IL-1 stimulation (Guo Y. et al., 2020). Deeper effects of autophagy on HA accumulation in GO remain to be investigated.

## 4 Autophagy in other eye diseases

Past studies have demonstrated that autophagy can play a crucial role in preserving cellular homezostasis in most cases (Klionsky et al., 2021) and that it is associated with the pathogenesis of other eye diseases (Panigrahi et al., 2019; Ishikawa et al., 2021; Kumar and Jurkunas, 2021; Shim et al., 2021; Villarejo-Zori et al., 2021; Feng et al., 2022; Yan et al., 2022). Autophagy is intricately involved in the development of ocular diseases such as glaucoma, cataract, DR, and ARMD. Trabecular meshwork (TM) cells regulate aqueous outflow and intraocular pressure (IOP). To protect themselves from oxidative stress and maintain intracellular homeostasis, TM cells activate autophagy, a process that removes damaged proteins and organelles. However, when autophagy is insufficient, non-degradable substances accumulate in lysosomes and reduce their activity, thereby decreasing autophagic flux and glaucoma progressions (Porter et al., 2013). Shim et al. reported that primary cilia (PC) can modulate autophagy through AKT and SMAD2/3 pathways in trabecular meshwork cells. When PC is absent, the compensatory responses to high IOP is impaired, which increases the LC3-II protein levels in response to increased pressures challenge (Shim et al., 2021). This finding implies that PC-mediated autophagy can play a role in modulating IOP homeostasis. Under normal conditions, the autophagic activity can contribute to the maintenance of normal lens function and transparency (Costello et al., 2013). However, when the elimination of organelles in the lens fiber cytoplasm is disturbed, ROS increases and homeostasis within the lens is disrupted, which decreases lens transparency and results in cataract development (Zhou et al., 2016). TBC1 domain family member 20 (TBC1D20) is an important factor regulating autophagosome formation. It alters the autophagosome expression, resulting in the accumulation of autophagic material and pathological cataract development in mouse lenses (Sidjanin et al., 2016). In addition, the knocking out of Atg5 mice also reduced transparency in the cortical region of the lenses (Morishita et al., 2013). These findings indicated that autophagy regulates intracellular homeostasis, preserves cell integrity, and maintains the physical properties of lens tissues.

Autophagy normally acts as a self-defense system against damage to the retinal pigment epithelium (RPE) by removing damaged materials and organelles. However, excessive metabolic stress leads to dysfunctional autophagy (Volpe et al., 2018). Cell death (through apoptosis, necrosis, and autophagy) can be induced by reactive oxygen species (ROS) and inflammatory cytokines (ILs) secreted in response to hyperglycemia (Volpe et al., 2018). As a result, retinal impairment is associated with autophagy activity in diabetic patients (Adornetto et al., 2021). In fact, the onset and development of ARMD has been attributed to the dysfunction of autophagy of RPE cells. Past studies have demonstrated two different ARMD mouse models (the knockout of Sod2 and APOE4-HFC); the autophagy elevated in the early stages and declined in the later ARMD stages (Mitter et al., 2014). In addition, the loss of LAMP2 (lysosomal associated membrane protein 2) expression in RPE cells is a typical step involved in the pathogenesis of dry ARMD in humans. The knocking out of Lamp2 in mice led to accelerated aging and the development of ARMD-like diseases. This observation can be attributed to an increase in the formation of basal layer deposits in the retina (Notomi et al., 2019).

## 5 Conclusion

We reviewed the current understanding of the role of autophagy in the onset and development of GO. Orbital tissue homeostasis, development, and cellular survival are all reliant on autophagy (Mizushima et al., 2008). Both excessive or insufficient autophagy can induce the pathogenesis of GO. In most cases, autophagy is responsible for maintaining homeostasis by regulating metabolism and recycling the cellular components (Mizushima et al., 2008). Keeping a balance between defective and excessive autophagy is therefore essential for the critical pathogenesis in GO, as abnormal autophagy can lead to orbital inflammation, adipogenesis, and GAG accumulation. Nevertheless, further work is required to develop a holistic comprehension of autophagy. Several signaling pathways have been demonstrated to regulate autophagy, such as mTOR, AMPK, and ER stress signaling pathways. Thus, to establish better therapeutic targets for GO, we need to investigate the mechanisms that regulate autophagy. Maintaining ocular homeostasis is crucial, and autophagy modifies several pathological processes associated with GO. As multiple factors participate in autophagy regulation, its complete mechanism remain unknown. Potential therapeutic strategies for GO can thus be established with the help of future research into the interplay between autophagy and the pathogenesis of GO in inflammatory responses, adipogenesis, and GAG accumulation.

## References

[B1] Adornetto A. Gesualdo C. Laganà M. L. Trotta M. C. Rossi S. Russo R. (2021). Autophagy: A novel pharmacological target in diabetic retinopathy. Front. Pharmacol. 12, 695267. 10.3389/fphar.2021.695267 34234681 PMC8256993

[B2] Amaravadi R. K. Yu D. Lum J. J. Bui T. Christophorou M. A. Evan G. I. (2007). Autophagy inhibition enhances therapy-induced apoptosis in a Myc-induced model of lymphoma. J. Clin. Invest. 117, 326–336. 10.1172/JCI28833 17235397 PMC1765515

[B3] Aniszewski J. P. Valyasevi R. W. Bahn R. S. (2000). Relationship between disease duration and predominant orbital T cell subset in Graves' ophthalmopathy. J. Clin. Endocrinol. Metab. 85, 776–780. 10.1210/jcem.85.2.6333 10690890

[B4] Bahn R. S. (2003). Clinical review 157: Pathophysiology of graves' ophthalmopathy: The cycle of disease. J. Clin. Endocrinol. Metab. 88, 1939–1946. 10.1210/jc.2002-030010 12727937

[B5] Bahn R. S. (2015). Current insights into the pathogenesis of graves' ophthalmopathy. Horm. Metab. Res. 47, 773–778. 10.1055/s-0035-1555762 26361262

[B6] Bahn R. S. (2010). Graves' ophthalmopathy. N. Engl. J. Med. 362, 726–738. 10.1056/NEJMra0905750 20181974 PMC3902010

[B7] Baird L. Yamamoto M. Takahashi Y. Hishinuma E. Saigusa D. (2020). Geldanamycin-derived HSP90 inhibitors are synthetic lethal with NRF2. Mol. Cell Biol. 40, . 10.1128/MCB.00377-20 PMC7588872 32868290

[B8] Ban Y. Tomer Y. (2003). The contribution of immune regulatory and thyroid specific genes to the etiology of Graves' and Hashimoto's diseases. Autoimmunity 36, 367–379. 10.1080/08916930310001603037 14669944

[B9] Bartalena L. Kahaly G. J. Baldeschi L. Dayan C. M. Eckstein A. Marcocci C. (2021). The 2021 European Group on Graves' orbitopathy (EUGOGO) clinical practice guidelines for the medical management of Graves' orbitopathy. Eur. J. Endocrinol. 185, G43–g67. 10.1530/EJE-21-0479 34297684

[B10] Baskaran R. Poornima P. Priya L. B. Huang C. Y. Padma V. V. (2016). Neferine prevents autophagy induced by hypoxia through activation of Akt/mTOR pathway and Nrf2 in muscle cells. Biomed. Pharmacother. 83, 1407–1413. 10.1016/j.biopha.2016.08.063 27583981

[B11] Bifulco M. Ciaglia E. (2016). Statin reduces orbitopathy risk in patients with Graves' disease by modulating apoptosis and autophagy activities. Endocrine 53, 649–650. 10.1007/s12020-015-0762-z 26438397

[B12] Cocco S. Leone A. Piezzo M. Caputo R. Di Lauro V. Di Rella F. (2020). Targeting autophagy in breast cancer. Int. J. Mol. Sci. 21, 7836. 10.3390/ijms21217836 33105796 PMC7660056

[B13] Costello M. J. Brennan L. A. Basu S. Chauss D. Mohamed A. Gilliland K. O. (2013). Autophagy and mitophagy participate in ocular lens organelle degradation. Exp. Eye Res. 116, 141–150. 10.1016/j.exer.2013.08.017 24012988 PMC3856666

[B14] Croce K. R. Yamamoto A. (2019). A role for autophagy in Huntington's disease. Neurobiol. Dis. 122, 16–22. 10.1016/j.nbd.2018.08.010 30149183 PMC6364695

[B15] Degenhardt K. Mathew R. Beaudoin B. Bray K. Anderson D. Chen G. (2006). Autophagy promotes tumor cell survival and restricts necrosis, inflammation, and tumorigenesis. Cancer Cell 10, 51–64. 10.1016/j.ccr.2006.06.001 16843265 PMC2857533

[B16] Ehrhart-Bornstein M. Lamounier-Zepter V. Schraven A. Langenbach J. Willenberg H. S. Barthel A. (2003). Human adipocytes secrete mineralocorticoid-releasing factors. Proc. Natl. Acad. Sci. U. S. A. 100, 14211–14216. 10.1073/pnas.2336140100 14614137 PMC283571

[B17] Fang S. Huang Y. Liu X. Zhong S. Wang N. Zhao B. (2018). Interaction between CCR6+ Th17 cells and CD34+ fibrocytes promotes inflammation: Implications in graves' orbitopathy in Chinese population. Invest. Ophthalmol. Vis. Sci. 59, 2604–2614. 10.1167/iovs.18-24008 29847667

[B18] Feng L. Liang L. Zhang S. Yang J. Yue Y. Zhang X. (2022). HMGB1 downregulation in retinal pigment epithelial cells protects against diabetic retinopathy through the autophagy-lysosome pathway. Autophagy 18, 320–339. 10.1080/15548627.2021.1926655 34024230 PMC8942416

[B19] Fernández-Albarral J. A. de Julián-López E. Soler-Domínguez C. de Hoz R. López-Cuenca I. Salobrar-García E. (2021). The Role of Autophagy in Eye Diseases, 11.Life (Basel) 10.3390/life11030189 PMC7997177 33673657

[B20] Guo J. Y. Chiu C. H. Wang M. J. Li F. A. Chen J. Y. (2020a). Proteoglycan serglycin promotes non-small cell lung cancer cell migration through the interaction of its glycosaminoglycans with CD44. J. Biomed. Sci. 27, 2. 10.1186/s12929-019-0600-3 31898491 PMC6939340

[B21] Guo Y. Li H. Chen X. Yang H. Guan H. He X. (2020b). Novel roles of chloroquine and hydroxychloroquine in graves' orbitopathy therapy by targeting orbital fibroblasts. J. Clin. Endocrinol. Metab. 105, 1906–1917. 10.1210/clinem/dgaa161 32249902 PMC7183395

[B22] Hara T. Nakamura K. Matsui M. Yamamoto A. Nakahara Y. Suzuki-Migishima R. (2006). Suppression of basal autophagy in neural cells causes neurodegenerative disease in mice. Nature 441, 885–889. 10.1038/nature04724 16625204

[B23] Harris J. Master S. S. De Haro S. A. Delgado M. Roberts E. A. Hope J. C. (2009). Th1-Th2 polarisation and autophagy in the control of intracellular mycobacteria by macrophages. Vet. Immunol. Immunopathol. 128, 37–43. 10.1016/j.vetimm.2008.10.293 19026454 PMC2789833

[B24] Hiromatsu Y. Yang D. Bednarczuk T. Miyake I. Nonaka K. Inoue Y. (2000). Cytokine profiles in eye muscle tissue and orbital fat tissue from patients with thyroid-associated ophthalmopathy. J. Clin. Endocrinol. Metab. 85, 1194–1199. 10.1210/jcem.85.3.6433 10720061

[B25] Holczer M. Hajdú B. Lőrincz T. Szarka A. Bánhegyi G. Kapuy O. (2019). A double negative feedback loop between mTORC1 and AMPK kinases guarantees precise autophagy induction upon cellular stress. Int. J. Mol. Sci. 20, 5543. 10.3390/ijms20225543 31703252 PMC6888297

[B26] Ichimura Y. Waguri S. Sou Y. S. Kageyama S. Hasegawa J. Ishimura R. (2013). Phosphorylation of p62 activates the Keap1-Nrf2 pathway during selective autophagy. Mol. Cell 51, 618–631. 10.1016/j.molcel.2013.08.003 24011591

[B27] Inami Y. Waguri S. Sakamoto A. Kouno T. Nakada K. Hino O. (2011). Persistent activation of Nrf2 through p62 in hepatocellular carcinoma cells. J. Cell Biol. 193, 275–284. 10.1083/jcb.201102031 21482715 PMC3080263

[B28] Ishikawa M. Takaseki S. Yoshitomi T. Covey D. F. Zorumski C. F. Izumi Y. (2021). The neurosteroid allopregnanolone protects retinal neurons by effects on autophagy and GABRs/GABA(A) receptors in rat glaucoma models. Autophagy 17, 743–760. 10.1080/15548627.2020.1731270 32070183 PMC8032250

[B29] Jaggi U. Matundan H. H. Lee D. H. Ghiasi H. (2022). Blocking autophagy in M1 macrophages enhances virus replication and eye disease in ocularly infected transgenic mice. J. Virol. 96, e0140122. 10.1128/jvi.01401-22 36286481 PMC9645210

[B30] Jang S. Y. Chae M. K. Lee J. H. Lee E. J. Yoon J. S. (2016). Role of miR-146a in the regulation of inflammation in an *in vitro* model of graves' orbitopathy. Invest. Ophthalmol. Vis. Sci. 57, 4027–4034. 10.1167/iovs.16-19213 27494344

[B31] Jang S. Y. Park S. J. Chae M. K. Lee J. H. Lee E. J. Yoon J. S. (2018). Role of microRNA-146a in regulation of fibrosis in orbital fibroblasts from patients with Graves' orbitopathy. Br. J. Ophthalmol. 102, 407–414. 10.1136/bjophthalmol-2017-310723 29101123

[B32] Jiang G. M. Tan Y. Wang H. Peng L. Chen H. T. Meng X. J. (2019). The relationship between autophagy and the immune system and its applications for tumor immunotherapy. Mol. Cancer 18, 17. 10.1186/s12943-019-0944-z 30678689 PMC6345046

[B33] Kaushik S. Cuervo A. M. (2018). The coming of age of chaperone-mediated autophagy. Nat. Rev. Mol. Cell Biol. 19, 365–381. 10.1038/s41580-018-0001-6 29626215 PMC6399518

[B34] Kim J. Kundu M. Viollet B. Guan K. L. (2011). AMPK and mTOR regulate autophagy through direct phosphorylation of Ulk1. Nat. Cell Biol. 13, 132–141. 10.1038/ncb2152 21258367 PMC3987946

[B35] Klionsky D. J. Petroni G. Amaravadi R. K. Baehrecke E. H. Ballabio A. Boya P. (2021). Autophagy in major human diseases. Embo J. 40, e108863. 10.15252/embj.2021108863 34459017 PMC8488577

[B36] Ko J. Kim J. Y. Kyoung Chae M. Jig Lee E. Sook Yoon J. (2021). PERK mediates oxidative stress and adipogenesis in Graves' orbitopathy pathogenesis. J. Mol. Endocrinol. 66, 313–323. 10.1530/JME-21-0057 33870911

[B37] Komatsu M. Waguri S. Chiba T. Murata S. Iwata J. Tanida I. (2006). Loss of autophagy in the central nervous system causes neurodegeneration in mice. Nature 441, 880–884. 10.1038/nature04723 16625205

[B38] Kumar S. Bahn R. S. (2003). Relative overexpression of macrophage-derived cytokines in orbital adipose tissue from patients with graves' ophthalmopathy. J. Clin. Endocrinol. Metab. 88, 4246–4250. 10.1210/jc.2003-030380 12970294

[B39] Kumar V. Jurkunas U. V. (2021). Mitochondrial dysfunction and mitophagy in fuchs endothelial corneal dystrophy. Cells 10. 10.3390/cells10081888 PMC8392447 34440658

[B40] Kuriyan A. E. Woeller C. F. O'Loughlin C. W. Phipps R. P. Feldon S. E. (2013). Orbital fibroblasts from thyroid eye disease patients differ in proliferative and adipogenic responses depending on disease subtype. Invest. Ophthalmol. Vis. Sci. 54, 7370–7377. 10.1167/iovs.13-12741 24135759 PMC3823547

[B41] Łacheta D. Miśkiewicz P. Głuszko A. Nowicka G. Struga M. Kantor I. (2019). Immunological aspects of graves' ophthalmopathy. Biomed. Res. Int. 2019, 7453260. 10.1155/2019/7453260 31781640 PMC6875285

[B42] Lau A. Wang X. J. Zhao F. Villeneuve N. F. Wu T. Jiang T. (2010). A noncanonical mechanism of Nrf2 activation by autophagy deficiency: Direct interaction between Keap1 and p62. Mol. Cell Biol. 30, 3275–3285. 10.1128/MCB.00248-10 20421418 PMC2897585

[B43] Lehmann G. M. Feldon S. E. Smith T. J. Phipps R. P. (2008). Immune mechanisms in thyroid eye disease. Thyroid 18, 959–965. 10.1089/thy.2007.0407 18752427 PMC2795569

[B44] Li H. Gao L. Min J. Yang Y. Zhang R. (2021). Neferine suppresses autophagy-induced inflammation, oxidative stress and adipocyte differentiation in Graves' orbitopathy. J. Cell Mol. Med. 25, 1949–1957. 10.1111/jcmm.15931 33443817 PMC7882929

[B45] Li H. Yuan Y. Zhang Y. Zhang X. Gao L. Xu R. (2017). Icariin inhibits AMPK-dependent autophagy and adipogenesis in adipocytes *in vitro* and in a model of graves' orbitopathy *in vivo* . Front. Physiol. 8, 45. 10.3389/fphys.2017.00045 28243204 PMC5303717

[B46] Li H. Zhang Y. Min J. Gao L. Zhang R. Yang Y. (2018). Astragaloside IV attenuates orbital inflammation in Graves' orbitopathy through suppression of autophagy. Inflamm. Res. 67, 117–127. 10.1007/s00011-017-1100-0 29127443

[B47] Li Y. Chen Y. (2019). AMPK and autophagy. Adv. Exp. Med. Biol. 1206, 85–108. 31776981 10.1007/978-981-15-0602-4_4

[B48] Lizama B. N. Chu C. T. (2021). Neuronal autophagy and mitophagy in Parkinson's disease. Mol. Asp. Med. 82, 100972. 10.1016/j.mam.2021.100972 PMC8665948 34130867

[B49] Longo C. M. Higgins P. J. (2019). Molecular biomarkers of Graves' ophthalmopathy. Exp. Mol. Pathol. 106, 1–6. 10.1016/j.yexmp.2018.11.004 30414981 PMC6381289

[B50] Maiuri M. C. Zalckvar E. Kimchi A. Kroemer G. (2007). Self-eating and self-killing: Crosstalk between autophagy and apoptosis. Nat. Rev. Mol. Cell Biol. 8, 741–752. 10.1038/nrm2239 17717517

[B51] Meyer zu Hörste M. Ströher E. Berchner-Pfannschmidt U. Schmitz-Spanke S. Pink M. Göthert J. R. (2011). A novel mechanism involved in the pathogenesis of graves ophthalmopathy (GO): Clathrin is a possible targeting molecule for inhibiting local immune response in the orbit. J. Clin. Endocrinol. Metab. 96, E1727–E1736. 10.1210/jc.2011-1156 21917865

[B52] Mimura L. Y. Villares S. M. Monteiro M. L. Guazzelli I. C. Bloise W. (2003). Peroxisome proliferator-activated receptor-gamma gene expression in orbital adipose/connective tissues is increased during the active stage of Graves' ophthalmopathy. Thyroid 13, 845–850. 10.1089/105072503322401032 14588098

[B53] Mitter S. K. Song C. Qi X. Mao H. Rao H. Akin D. (2014). Dysregulated autophagy in the RPE is associated with increased susceptibility to oxidative stress and AMD. Autophagy 10, 1989–2005. 10.4161/auto.36184 25484094 PMC4502658

[B54] Mizushima N. Klionsky D. J. (2007). Protein turnover via autophagy: Implications for metabolism. Annu. Rev. Nutr. 27, 19–40. 10.1146/annurev.nutr.27.061406.093749 17311494

[B55] Mizushima N. Levine B. (2020). Autophagy in human diseases. N. Engl. J. Med. 383, 1564–1576. 10.1056/NEJMra2022774 33053285

[B56] Mizushima N. Levine B. Cuervo A. M. Klionsky D. J. (2008). Autophagy fights disease through cellular self-digestion. Nature 451, 1069–1075. 10.1038/nature06639 18305538 PMC2670399

[B57] Morgan-Bathke M. Lin H. H. Chibly A. M. Zhang W. Sun X. Chen C. H. (2013). Deletion of ATG5 shows a role of autophagy in salivary homeostatic control. J. Dent. Res. 92, 911–917. 10.1177/0022034513499350 23884556 PMC3775371

[B58] Morishita H. Eguchi S. Kimura H. Sasaki J. Sakamaki Y. Robinson M. L. (2013). Deletion of autophagy-related 5 (Atg5) and Pik3c3 genes in the lens causes cataract independent of programmed organelle degradation. J. Biol. Chem. 288, 11436–11447. 10.1074/jbc.M112.437103 23479732 PMC3630873

[B59] Niederkorn J. Y. (2006). See no evil, hear no evil, do no evil: The lessons of immune privilege. Nat. Immunol. 7, 354–359. 10.1038/ni1328 16550198

[B60] Nishida Y. Tian S. Isberg B. Hayashi O. Tallstedt L. Lennerstrand G. (2002). Significance of orbital fatty tissue for exophthalmos in thyroid-associated ophthalmopathy. Graefes Arch. Clin. Exp. Ophthalmol. 240, 515–520. 10.1007/s00417-002-0498-3 12136278

[B61] Notomi S. Ishihara K. Efstathiou N. E. Lee J. J. Hisatomi T. Tachibana T. (2019). Genetic LAMP2 deficiency accelerates the age-associated formation of basal laminar deposits in the retina. Proc. Natl. Acad. Sci. U. S. A. 116, 23724–23734. 10.1073/pnas.1906643116 31699817 PMC6876195

[B62] Panigrahi T. Shivakumar S. Shetty R. D'Souza S. Nelson E. J. R. Sethu S. (2019). Trehalose augments autophagy to mitigate stress induced inflammation in human corneal cells. Ocul. Surf. 17, 699–713. 10.1016/j.jtos.2019.08.004 31412290

[B63] Park S. Park D. Y. Kim J. Woo K. I. Kim Y. D. Han J. (2020). Enhanced orbital adipogenesis in a mouse model of T-cell-mediated autoimmunity, zymosan A-treated SKG mice: Implications for Graves' ophthalmopathy. Sci. Rep. 10, 7329. 10.1038/s41598-020-64402-9 32355208 PMC7193596

[B64] Potgieser P. W. Wiersinga W. M. Regensburg N. I. Mourits M. P. (2015). Some studies on the natural history of graves' orbitopathy: Increase in orbital fat is a rather late phenomenon. Eur. J. Endocrinol. 173, 149–153. 10.1530/EJE-14-1140 26142100

[B65] Prabhakar B. S. Bahn R. S. Smith T. J. (2003). Current perspective on the pathogenesis of Graves' disease and ophthalmopathy. Endocr. Rev. 24, 802–835. 10.1210/er.2002-0020 14671007

[B66] Rodriguez A. Duran A. Selloum M. Champy M. F. Diez-Guerra F. J. Flores J. M. (2006). Mature-onset obesity and insulin resistance in mice deficient in the signaling adapter p62. Cell Metab. 3, 211–222. 10.1016/j.cmet.2006.01.011 16517408

[B67] Rosen E. D. MacDougald O. A. (2006). Adipocyte differentiation from the inside out. Nat. Rev. Mol. Cell Biol. 7, 885–896. 10.1038/nrm2066 17139329

[B68] Santeford A. Wiley L. A. Park S. Bamba S. Nakamura R. Gdoura A. (2016). Impaired autophagy in macrophages promotes inflammatory eye disease. Autophagy 12, 1876–1885. 10.1080/15548627.2016.1207857 27463423 PMC5066937

[B69] Schrijver B. Kooiman M. A. Kasteleijn E. van Holten-Neelen C. Virakul S. Paridaens D. (2019). Basic fibroblast growth factor induces adipogenesis in orbital fibroblasts: Implications for the pathogenesis of graves' orbitopathy. Thyroid 29, 395–404. 10.1089/thy.2018.0544 30724135

[B70] Shim M. S. Nettesheim A. Dixon A. Liton P. B. (2021). Primary cilia and the reciprocal activation of AKT and SMAD2/3 regulate stretch-induced autophagy in trabecular meshwork cells. Proc. Natl. Acad. Sci. U. S. A. 118, e2021942118. 10.1073/pnas.2021942118 33753495 PMC8020776

[B71] Sidjanin D. J. Park A. K. Ronchetti A. Martins J. Jackson W. T. (2016). TBC1D20 mediates autophagy as a key regulator of autophagosome maturation. Autophagy 12, 1759–1775. 10.1080/15548627.2016.1199300 27487390 PMC5079675

[B72] Smith T. J. Bahn R. S. Gorman C. A. (1989). Connective tissue, glycosaminoglycans, and diseases of the thyroid. Endocr. Rev. 10, 366–391. 10.1210/edrv-10-3-366 2673756

[B73] Smith T. J. Janssen J. (2019). Insulin-like growth factor-I receptor and thyroid-associated ophthalmopathy. Endocr. Rev. 40, 236–267. 10.1210/er.2018-00066 30215690 PMC6338478

[B74] Smith T. J. Kahaly G. J. Ezra D. G. Fleming J. C. Dailey R. A. Tang R. A. (2017). Teprotumumab for thyroid-associated ophthalmopathy. N. Engl. J. Med. 376, 1748–1761. 10.1056/NEJMoa1614949 28467880 PMC5718164

[B75] Smith T. J. Koumas L. Gagnon A. Bell A. Sempowski G. D. Phipps R. P. (2002). Orbital fibroblast heterogeneity may determine the clinical presentation of thyroid-associated ophthalmopathy. J. Clin. Endocrinol. Metab. 87, 385–392. 10.1210/jcem.87.1.8164 11788681

[B76] Sorisky A. Pardasani D. Gagnon A. Smith T. J. (1996). Evidence of adipocyte differentiation in human orbital fibroblasts in primary culture. J. Clin. Endocrinol. Metab. 81, 3428–3431. 10.1210/jcem.81.9.8784110 8784110

[B77] Suzuki S. Morimoto S. Fujishiro M. Kawasaki M. Hayakawa K. Miyashita T. (2015). Inhibition of the insulin-like growth factor system is a potential therapy for rheumatoid arthritis. Autoimmunity 48, 251–258. 10.3109/08916934.2014.976631 25352179

[B78] Tsushima H. Morimoto S. Fujishiro M. Yoshida Y. Hayakawa K. Hirai T. (2017). Kinase inhibitors of the IGF-1R as a potential therapeutic agent for rheumatoid arthritis. Autoimmunity 50, 329–335. 10.1080/08916934.2017.1344970 28682648

[B79] Villarejo-Zori B. Jiménez-Loygorri J. I. Zapata-Muñoz J. Bell K. Boya P. (2021). New insights into the role of autophagy in retinal and eye diseases. Mol. Asp. Med. 82, 101038. 10.1016/j.mam.2021.101038 34620506

[B80] Volpe C. M. O. Villar-Delfino P. H. Dos Anjos P. M. F. Nogueira-Machado J. A. (2018). Cellular death, reactive oxygen species (ROS) and diabetic complications. Cell Death Dis. 9, 119. 10.1038/s41419-017-0135-z 29371661 PMC5833737

[B81] Wang L. Klionsky D. J. Shen H. M. (2022a). The emerging mechanisms and functions of microautophagy. Nat. Rev. Mol. Cell Biol. 24, 186–203. 10.1038/s41580-022-00529-z 36097284

[B82] Wang W. Yu Z. Y. Song R. H. He S. T. Shi L. F. Zhang J. A. (2022b). Polymorphisms of ATG5 gene are associated with autoimmune thyroid diseases, especially thyroid eye disease. J. Immunol. Res. 2022, 3881417. 10.1155/2022/3881417 35518570 PMC9064513

[B83] Wang Y. Smith T. J. (2014). Current concepts in the molecular pathogenesis of thyroid-associated ophthalmopathy. Invest. Ophthalmol. Vis. Sci. 55, 1735–1748. 10.1167/iovs.14-14002 24651704 PMC3968932

[B84] Wu X. Liu Z. Yu X. Y. Xu S. Luo J. (2021). Autophagy and cardiac diseases: Therapeutic potential of natural products. Med. Res. Rev. 41, 314–341. 10.1002/med.21733 32969064

[B85] Yan X. Wu S. Liu Q. Cheng Y. Zhang J. Wang N. (2022). Myocilin gene mutation induced autophagy activation causes dysfunction of trabecular meshwork cells. Front. Cell Dev. Biol. 10, 900777. 10.3389/fcell.2022.900777 35615698 PMC9124892

[B86] Yang X. Pan X. Zhao X. Luo J. Xu M. Bai D. (2019). Autophagy and age-related eye diseases. Biomed. Res. Int. 2019, 5763658. 10.1155/2019/5763658 31950044 PMC6948295

[B87] Yoon J. S. Lee H. J. Chae M. K. Lee E. J. (2015). Autophagy is involved in the initiation and progression of Graves' orbitopathy. Thyroid 25, 445–454. 10.1089/thy.2014.0300 25687157

[B88] Yoon Y. Chae M. K. Lee E. J. Yoon J. S. (2020). 4-Methylumbelliferone suppresses hyaluronan and adipogenesis in primary cultured orbital fibroblasts from Graves' orbitopathy. Graefes Arch. Clin. Exp. Ophthalmol. 258, 1095–1102. 10.1007/s00417-019-04528-3 31900640

[B89] Yoshida M. Minagawa S. Araya J. Sakamoto T. Hara H. Tsubouchi K. (2019). Involvement of cigarette smoke-induced epithelial cell ferroptosis in COPD pathogenesis. Nat. Commun. 10, 3145. 10.1038/s41467-019-10991-7 31316058 PMC6637122

[B90] Yu L. Chen Y. Tooze S. A. (2018). Autophagy pathway: Cellular and molecular mechanisms. Autophagy 14, 207–215. 10.1080/15548627.2017.1378838 28933638 PMC5902171

[B91] Zhang P. Zhu H. (2022). Cytokines in thyroid-associated ophthalmopathy. J. Immunol. Res. 2022, 2528046. 10.1155/2022/2528046 36419958 PMC9678454

[B92] Zhang Y. Goldman S. Baerga R. Zhao Y. Komatsu M. Jin S. (2009). Adipose-specific deletion of autophagy-related gene 7 (atg7) in mice reveals a role in adipogenesis. Proc. Natl. Acad. Sci. U. S. A. 106, 19860–19865. 10.1073/pnas.0906048106 19910529 PMC2785257

[B93] Zhang Z. Yang X. Song Y. Q. Tu J. (2021). Autophagy in Alzheimer's disease pathogenesis: Therapeutic potential and future perspectives. Ageing Res. Rev. 72. 10.1016/j.arr.2021.101464 101464 34551326

[B94] Zhao H. Wang Y. Qiu T. Liu W. Yao P. (2020). Autophagy, an important therapeutic target for pulmonary fibrosis diseases. Clin. Chim. Acta 502, 139–147. 10.1016/j.cca.2019.12.016 31877297

[B95] Zhou J. Yao K. Zhang Y. Chen G. Lai K. Yin H. (2016). Thioredoxin binding protein-2 regulates autophagy of human lens epithelial cells under oxidative stress via inhibition of akt phosphorylation. Oxid. Med. Cell Longev. 2016, 4856431. 10.1155/2016/4856431 27656263 PMC5021881

